# Use of newly synthetized magnetic Fe_3_O_4_ nanoparticles modified with hexadecyl trimethyl ammonium bromide for the sensitive analysis of antidepressant drugs, duloxetine and vilazodone in wastewater and urine samples

**DOI:** 10.1039/d3ra02442c

**Published:** 2023-07-05

**Authors:** Halil Ibrahim Ulusoy, Ummugulsum Polat, Songül Ulusoy

**Affiliations:** a Department of Analytical Chemistry, Faculty of Pharmacy, Sivas Cumhuriyet University Sivas 58140 Türkiye hiulusoy@yahoo.com +90 346 219 16 34 +90 346 487 3905; b Department of Pharmacy, Vocational School of Health Service, Cumhuriyet University Sivas 58140 Türkiye

## Abstract

A new enrichment and determination method involving HPLC-DAD analysis following magnetic solid-phase extraction (MSPE) was developed to detect trace amounts of two antidepressant drugs, namely, duloxetine (DUL) and vilazodone (VIL). In this study, a solid-phase sorbent was newly synthesized for use in the MSPE and its characterization was carried out by scanning electron microscopy (SEM), Fourier transform infrared (FTIR) spectroscopy, Raman spectroscopy and X-ray diffraction (XRD) techniques. In this proposed method, DUL and VIL molecules were enriched using newly synthesized magnetic-based nanoparticles in the presence of pH 10.0 buffer and desorbed with acetonitrile to a smaller volume prior to chromatographic determinations. After experimental variables were optimized, the VIL and DUL molecules were analyzed at wavelengths of 228 nm for DUL and 238 nm for VIL with isocratic elution of methanol, trifluoroacetic acid (TFA) (0.1%), and acetonitrile (10 : 60 : 30). The detection limits obtained under optimized conditions were 1.48 ng mL^−1^ and 1.43 ng mL^−1^, respectively. The %RSD values were found to be lower than 3.50% with model solutions containing 100 ng mL^−1^ (N:5). Finally, the developed method was successfully applied to wastewater samples and simulated urine samples, and quantitative results were obtained in the recovery experiments.

## Abstract 2

A new enrichment and determination method involving HPLC-DAD analysis following magnetic solid-phase extraction (MSPE) was developed to detect trace amounts of two antidepressant drugs, namely, duloxetine (DUL) and vilazodone (VIL).

## Graphical abstract

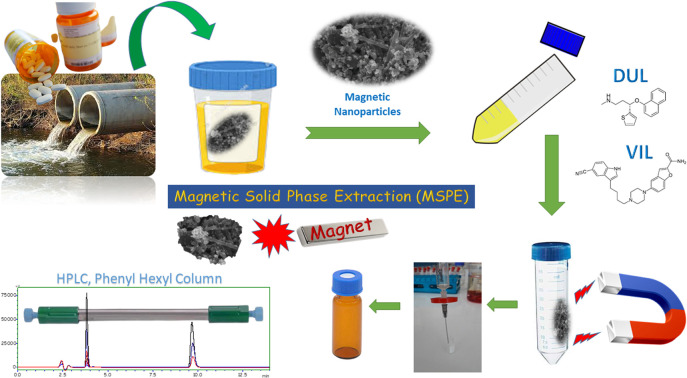

## 1. Introduction

Depression is a major health problem that is chronic, recurrent and accompanied by cognitive, psychomotor and psychophysiological disorders, causing serious loss of work power and ability.^1^ Clinically, depression is an emotional state, that is, when a person is in a constantly sad, distressed, or frequently changing mood for a certain period of time, it may result in slowing down of thoughts, speech and movements, physiological disorders, stagnation, feeling worthless and inferior, weakness, reluctance, and pessimism. It is a syndrome related to emotions and thoughts.^2^

Antidepressant drugs are widely and effectively used in the treatment of many mood disorders, especially depression, psychotic disorders and substance-dependent mood disorders. It is also used in the treatment of sleeplessness, anxiety and eating disorders, pain syndromes, arrhythmias and some immune dysfunctions.^3^ Unnecessary use of antidepressant drugs is increasing every year. This means that economic loss is also increasing due to misuse of drugs. Moreover, unconscious use causes heart rhythm problems, muscle stiffness, changes in blood pressure if they are not taken in recommended time and doses.^4^

Adequate dose and duration are very important in treatment with antidepressant drugs. An important feature of almost all antidepressant drugs is that efficacy begins within 1–3 weeks. During this period, sleep, appetite, mood and social activities begin to improve. A period of 4–6 weeks is mandatory to decide whether the treatment is effective or not.^5^ If there is no positive response within this period, the same drug should not be insisted on, and a drug from another group or a drug with a different mechanism of action should be selected. Priority should be given to the antidepressant drug that the person or a family member is using or has been treated with before. In some of the patients who seem to be resistant to treatment, it is observed that adequate doses and durations are not followed. Subclinical hypothyroidism and cerebrovascular events such as stroke may accompany in most of the cases that comply with these conditions. In cases where there is a response to treatment, the treatment should be continued for 6–12 months after improvement. After a 6–12 months maintenance period, drugs should be tapered and discontinued within months.^6^

As with many active drug ingredients, it is important to analyze low concentrations of such molecules after use in order to follow their therapeutic effects and excretion products in urine. It is a big challenge to determine drug residues in biological samples due to their trace concentrations and high concentrations of matrix ingredients. Most of the conventional instrumental systems including chromatographic, spectroscopic, and electro-analytical methods do not have the ability for direct determination of trace molecules. They generally need a pre-treatment procedure for the separation of matrix components and pre-concentration of target molecules.

Currently, the monitoring of low concentrations of antidepressant drugs, especially in biological samples, with high sensitivity, good selectivity and high efficiency is mostly performed by liquid chromatography-mass spectrometry (LC-MS)-based analysis methods.^7^ Other analytical methods including high-performance liquid chromatography with fluorescence detection (HPLC-FD), HPLC with ultraviolet detection (HPLC-UV), gas chromatography-MS (GC-MS) are also available with higher detection limits. Because a sample with a complex matrix has many distinct peaks and cannot be separated effectively, conventional liquid chromatography alone can no longer meet the detection sensitivity requirements.^8^ When the GC-MS detection of these types of drugs is attempted, it is necessary to prepare a derivative of the target molecules which make it unsuitable for the analysis of many biological samples, and hence, it is not preferred.^9^ Liquid chromatography-based methods, which are more suitable approaches for biological samples such as blood, urine, milk and tissue fluid, can become very useful with an appropriate sample preparation approach.^10^

Sample preparation methods provide both enrichment as they the ability to increase the concentration of target molecules to a certain level and separation as they remove most species that have the potential to disrupt the analysis in the environment. Among such separation and enrichment methods, one of the most widely used methods in recent years is magnetic solid-phase extraction.^7,11^ This method has gained growing interest as solid-phase supports specific to the target species are used.

Solid-phase extraction-based methods need revisions to accelerate the procedure, enhance the sensitivity, and increase the selectivity. Therefore, scientists try to develop new materials for SPE. Nowadays, magnetic solid-phase extraction (MSPE) is one of the most potential methods. One of the biggest problems in batch-type solid-phase extraction experiments is the separation of solid phase from aquatic solutions. In the MSPE, a magnetic sorbent was synthesized and used to facilitate phase separation using an external magnet, mostly neodymium.^12,13^

In this study, a magnetic solid-phase extraction method was developed for the determination of residues of two selected antidepressant drugs, namely, duloxetine and vilazodone in wastewater and urine samples prior to HPLC-DAD analysis. The synthesis and characterization of the new magnetic sorbent was carried out in detail. The optimization of the MSPE procedure and partial validation of total analytical methodology was done by using model solutions, and finally, the method was successively applied to real samples.

## 2. Materials and methods

### 2.1. Instrumentation

The characterization of the synthesized magnetic nanoparticles was carried out by Raman spectroscopy, X-ray diffraction spectroscopy and scanning electron microscopy. The Raman spectra of the nanomaterials were recorded using a Raman Spectrophotometer (WITEC alpha 300M + micro-Raman system, Germany) with a 532 nm laser source. X-ray diffraction spectra of the magnetic nanoparticles were recorded using a Bruker AXS D8 brand X-ray diffractometer. Scanning electron microscopy (SEM) and SEM mapping analysis were performed using a Zeiss Gemini 500 Field Emission Scanning Electron Microscope to elucidate the morphological structures of magnetic nanoparticles. Chromatographic analysis of fluoxetine and citalopram were performed using a Shimadzu (Prominence) HPLC (Kyoto, Japan) system. All separations and determinations were done using a phenyl hexyl column (Luna® 5 μm Phenyl-Hexyl 100 Å, 250 mm × 4.6 mm).

### 2.2. Chemicals and reagents

In this study, all the chemicals used are of 99.5% purity. A deionized water system with 18.2 MΩ cm resistivity was used to obtain deionized water (MES, MP Minipure Dest Up, Turkey). HPLC-grade acetonitrile and methanol were used for HPLC-DAD analysis (Sigma Aldrich, St. Louis, MO, USA). For HPLC analysis, we used a mixture of phosphate buffer solution (pH 3.0, 50 mM), methanol and acetonitrile (60 : 10 : 30) as the mobile phase. Stock solutions of duloxetine and vilazodone (Sigma Aldrich, St. Louis, MO, USA) were prepared in methanol and calibration mix standards in serial dilutions.

### 2.3. HPLC determination conditions for direct assays of duloxetine and vilazodone

In order to directly optimize the HPLC method, various mobile and stationary phases were investigated and all parameters were optimized step by step. The used HPLC method for the analysis of target drug molecules was developed in this study the first time. A phenyl hexyl column was chosen as the most suitable stationary phase for the effective separation of target molecules. For the mobile phase, various organic solvents and buffer solutions were tried by using them twice or thrice; in order to determine the ideal working phase compositions, aqueous solutions of organic-characterized working phases containing buffers at different pH values and different working phase compositions were used. The aim was to obtain the most accurate results for phase compositions by making many trials in isocratic and gradient elution modes.

**Fig. 1:**
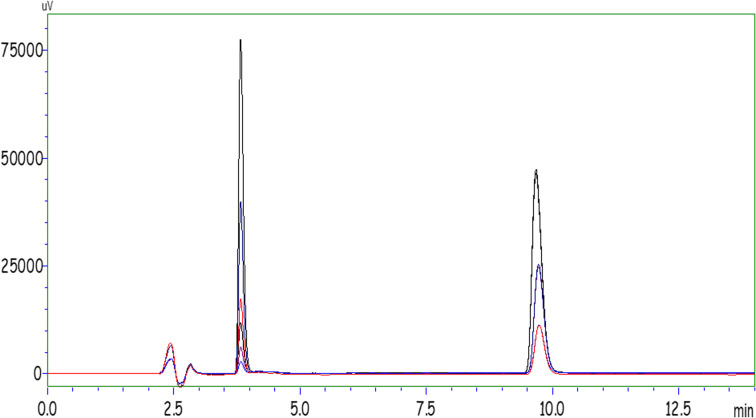
HPLC chromatogram of VIL and DUL molecules.

The ideal HPLC operating conditions obtained after optimization are given in Table 1 for duloxetine and vilazodone molecules. The chromatogram showing the ideal peaks of duloxetine and vilazodone obtained by the execution of the standards is shown in Fig. 1.

**Table 1:** HPLC Conditions for the proposed method

Parameter	Values
HPLC mode	Isocratic elution
Mobile phases	10% methanol
	60% triflouro acetic acid (0.1%)
	30% acetonitrile
Flow rate	1 mL min^−1^
Run time	15 min
Column	Phenyl hexyl, 250 × 50 mm, 5 μm
Column temperature	40 °C
Injection volume	10 μL

### 2.4. Synthesis of magnetic nanoparticles (Fe_3_O_4_@HDTMABr)

Synthesis of magnetic sorbents is a well-known procedure reported in the literature. Our research group has already used this procedure in previous studies with a few modifications.^14,15^ Briefly, iron salts (FeSO_4_·4H_2_O and FeCl_3_·6H_2_O) were dissolved in 50 mL of 0.1 M HCl, respectively. Then, 100 mL of ethyl alcohol: water mixture (1 : 1) was added to the solution under stirring at 600 rpm in the presence of nitrogen gas for the inert medium. The formation of Fe_3_O_4_ particles was started by adding 20 mL ammonia to the solution drop by drop under stirring using a magnetic stirrer. The resulting black particles were obtained using an external magnet, washed with a certain proportion of water/methyl alcohol mixture and dried in an oven at 60 °C for 6 hours.

Then, 2.5 gram of the as-obtained material was dispersed in 100 mL of 50% ethanol solution containing 1 mL of NH_3_. Tetraethyl orthosilicate (TEOS) was added to form the first layer on the surface of particles. Then, 500 mg of hexadecyltrimethylammonium bromide (HDTMABr) in 100 mL of 30% ethanol solution was transferred to the solution for final modification of magnetic particles. The mixture was kept on a magnetic stirrer at 65 °C for 6 hours in order to complete the synthesis procedure. Finally, the magnetic particles were washed several times with an ethanol/water mixture and then left to dry overnight.

### 2.5. The proposed method based on magnetic solid-phase extraction

First, 20 mL of sample solution including target molecules in the range of 5.0–750.0 ng mL^−1^ was transferred to a 50 mL falcon tube. Then, 50 mg of Fe_3_O_4_@HDTMABr and 2 mL of pH 10 buffer was transferred and the final volume was made up to 50 mL. The tubes were put on an orbital shaker at 50 rpm for 30 min in order to increase the interaction between drug molecules and magnetic particles. The aquatic phase was removed using a pipet with the help of an external magnet. The desorption of drug molecules was carried out by adding 800 μL of acetonitrile and using a vortex for 40 s. The acetonitrile phase was taken in a usual injector and filtered through a 0.45 μm injector tip filter. Then, the samples were transferred to HPLC vials and their drug concentration was analyzed by HPLC-DAD.

### 2.6. Preparation of simulated urine samples and wastewater samples

The drugs studied in this study are among the most frequently observed molecules in wastewater and urine samples. For the application of the developed method, samples were prepared following the procedures reported in the literature and used in the application phase.

Waste water samples: the samples were immediately taken from the Sivas Municipality wastewater treatment plant inlet point. The samples were transferred in amber glass bottles and filtered through a 0.45 μm cellulose nitrate membrane. Subsequently, the pH of samples was adjusted to 3 to reduce the biological activity,^16^ and the samples were stored in the darkness at +4 °C until analysis.

Urine samples: in the application area of the developed method, the urine sample was obtained directly from a healthy volunteer who is not on any medication. The volunteer was informed about the nature of the study and experimental procedure.^17^

Simulated urine solution: first, 6.25 g urea, 0.27 g CaCl_2_·2H_2_O, 0.25 g NH_3_Cl, 0.4 g KCl, 0.35 g Na_2_SO_4_, 0.35 g KH_2_PO_4_, and 0.73 g NaCl were weighed and dissolved in distilled water and the volume was made up to 250 mL in a volumetric balloon. Then, the pH of this solution was adjusted to 6 with 0.1 M HCl solution. It was transferred to an amber colored bottle and stored at +4 °C.^18–20^

## 3. Results and discussions

### 3.1. Characterization of the magnetic nanoparticles

#### FTIR analysis

**Fig. 2:**
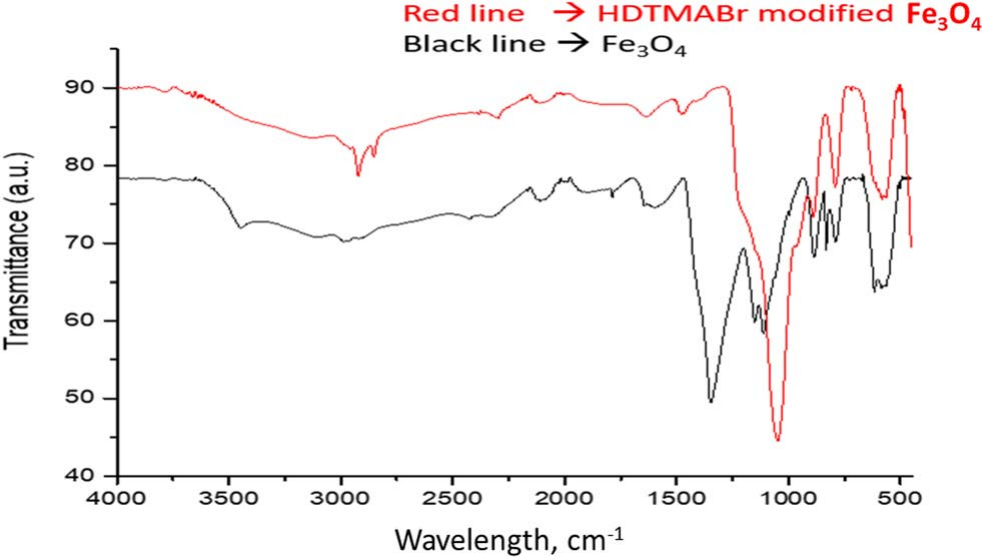
FTIR spectrum of the used magnetic particles.

The FT-IR spectra obtained for Fe_3_O_4_ and HDTMABr-coated Fe_3_O_4_ materials were analyzed in the wavelength range of 4000–450 cm^−1^ (Fig. 2). The peaks of the Fe_3_O_4_ material were at 616.82 and the peaks of Fe_3_O_4_@HDTMABr were at 787.52, 833.19, 886.9, 1112, 1153, 1643.1, 1787.2, and 2426.7, respectively. Moreover, the peaks appearing at 2986.8 and 3439.7 cm^−1^ wavelengths are also important to identify HDTMABr. It has been shown in literature studies that the band, which is seen as a broad spectrum at a wavelength of 3439.7 cm^−1^ in the wavelength range of 3300–3450 cm^−1^, is the –OH (hydroxyl)(H bond) tension band and that it can be encountered in pure Fe_3_O_4_ magnetite structures as the Fe–OH stretch spectrum confirmed.^21,22^

Free hydroxyl groups give a sharp peak between 3645 and 3610 cm^−1^. The fact that the spectrum seen at 3439.7 cm^−1^ is broader than expected is also interpreted as an intermolecular H bond accepted in the literature. The sharp peak seen at 2986 cm^−1^ wavelength is assigned to the C–H symmetric stretching vibration bands. It is thought that the spectrum observed at a wavelength of 2426.7 cm^−1^ may belong to the C–N stretching vibration originating from the atmosphere in the reaction medium. Furthermore, the spectra at 1643.1 and 1787.2 cm^−1^ wavelengths are assigned to the C=O and C–O stretching vibrations, respectively.^23,24^ In the wavelength range of 1500–1000 cm^−1^, which appears as the region where trend bands are seen, and in the wavelength range of 1000–450 cm^−1^ known as the fingerprint region, characteristic Fe_3_O_4_ peaks are observed at wavelengths of 616,82, 787,52, 833,19, 886,9, 1112, 1153 cm^−1^. The peaks of the HDTMABr-coated Fe_3_O_4_ material obtained after the reaction of the Fe_3_O_4_ nanomaterial with TEOS and hexadecylammonium bromide appeared at 582.01, 792.75, 884.26, 1047.4, 1477.6, 2297.4, 2853.4 and 2921.4 cm^−1^, respectively. Specific peaks of the TEOS intermediate reagent are seen at wavelengths of 793 and 1047.4 cm^−1^.^25^ In the wavelength range of 2970–2900 cm^−1^, the peak with a wavelength of 2921.4 cm^−1^, respectively, is ascribed to the C–H symmetrical stress-induced peak. Spectra at 582.01, 884.26, 1477.6, 2297.4, and 2853.4 cm^−1^ wavelengths are seen as characteristic peaks based on hexadecyltrimethylammonium bromide. It is clearly seen from the graphs of the overlaid spectra that most of the characteristic peaks of the starting product are suppressed in the product obtained after synthesis.

#### Raman analysis

**Fig. 3:**
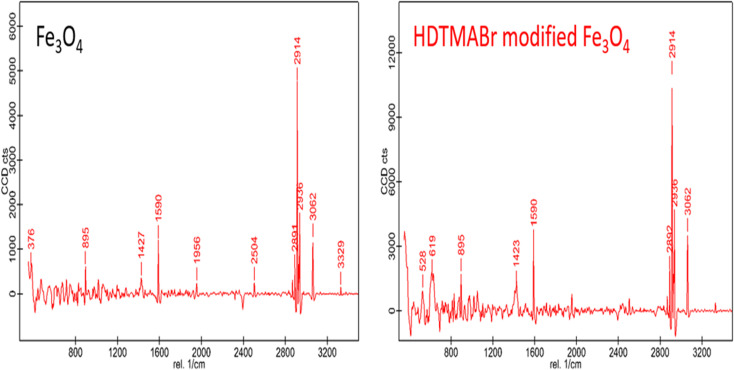
Raman spectrum of the used magnetic particles.

The Raman spectra of Fe_3_O_4_ and HDTMABr-coated Fe_3_O_4_ materials were obtained using a Raman spectrophotometer with a 532 nm laser as the excitation source, as can be seen in Fig. 3. The main peaks of the Fe_3_O_4_ material are Raman spectra with wavelengths of 895, 1427, 1590, 1956, 2504, 2914, 2936, 3062 and 3329 cm^−1^, respectively. A Raman peak at 895 cm^−1^ wavelength characterizes Fe_3_O_4_-based energy band gaps. The Raman spectrum with 1427 cm^−1^ wavelength in the 1410–1465 cm^−1^ wavelength range belongs to the energy bands formed as a result of the deformation of the –CH_2_ and –CH_3_ functional groups. A Raman peak with 1427 cm^−1^ wavelength seen in the 1410–1460 cm^−1^ wavelength range is ascribed to the deformation vibrations based on –CH_2_ and –CH_3_ functional groups. A Raman peak with a wavelength of 1590 cm^−1^ defines the atmospheric C–N and N–H vibrational stress bands. The peak observed at a wavelength of 1956 cm^−1^ is thought to be a Raman peak assigned to the asymmetric C=C vibrations. Intense Raman peaks seen at wavelengths of 2914 and 2936 cm^−1^ belong to energy bands based on C–H symmetric vibration. A Raman peak at 3062 cm^−1^ wavelength seen in the wavelength range of 3200 and 3330 cm^−1^ is a characteristic Raman peak for hydrogen bond shifted and expanded –NH (Fig. 3).

#### XRD

**Fig. 4:**
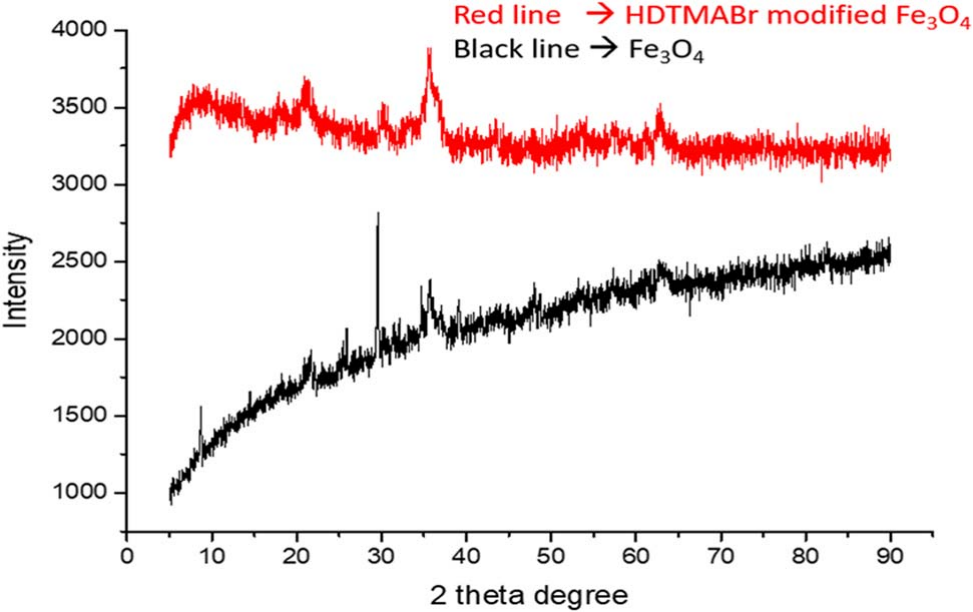
XRD spectrum of magnetic materials.

XRD (X-ray diffraction) patterns were acquired for Fe_3_O_4_ and HDTMABr-coated Fe_3_O_4_ nanomaterials. The diffraction peaks obtained for the Fe_3_O_4_ nanomaterial were 8.97°; 14.46°; 21.68°; 25.41; 29.72°; 34.71°; 47.86°; 57.29°; 62.74° (2theta). 21.68°; and 29.72°; XRD diffraction peaks of 34.71°, 57.29° and 62.74° are characteristic of Fe_3_O_4_ particles. The diffraction peaks of HDTMABr-coated Fe_3_O_4_ nanomaterials are seen in the XRD spectrum where the peaks in the 20–30° (2theta) range of Fe_3_O_4_ are suppressed due to the intermediate reagents converting the structure to amorphous (Fig. 4).

#### SEM analysis

**Fig. 5:**
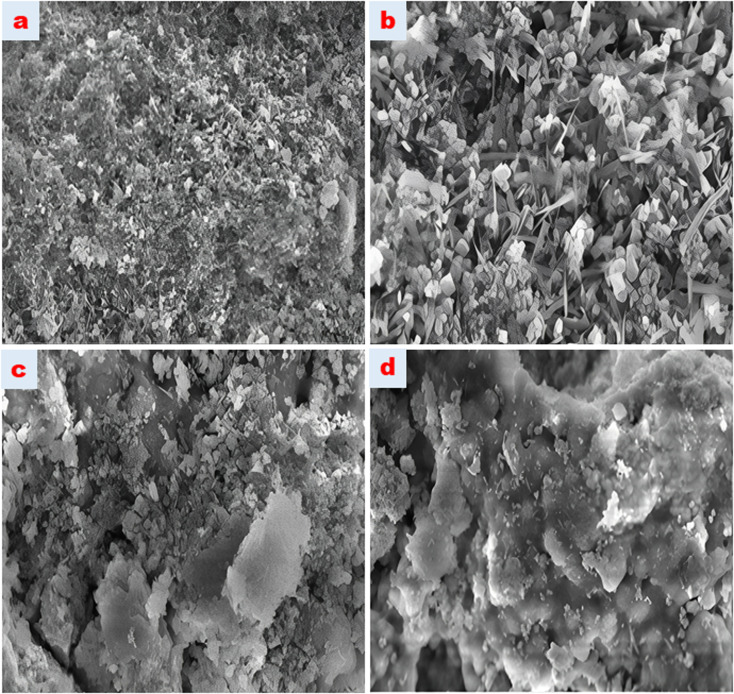
SEM images of the synthesized magnetic particle.

SEM images of Fe_3_O_4_ and HDTMABr-coated Fe_3_O_4_ nanomaterials were visualized using a scanning electron microscope, as shown in Fig. 5. At different magnifications (a: 20 k, b: 30 k, c: 50 k and d: 100 k), the images of nanomaterials are given as graphical representation. When the surface morphology of the HDTMABr-coated Fe_3_O_4_ nanomaterial is examined, a mixed form in elliptical and rod structures is observed. The homogeneous distribution of elliptical and rod-shaped magnetite in the structure was proved in the SEM analysis, and the amorphous structure was formed after the synthesis intermediate products were added.

### 3.2. Optimization steps of the proposed method

The synthetized magnetic particle was characterized in detail and used throughout optimization steps. Every step of MSPE was optimized by changing one parameter while the others are kept constant. All experiments were carried out in triplicate. The analytical signals were evaluated as peak areas of target molecules.

#### 3.2.1 Effect of pH

**Fig. 6:**
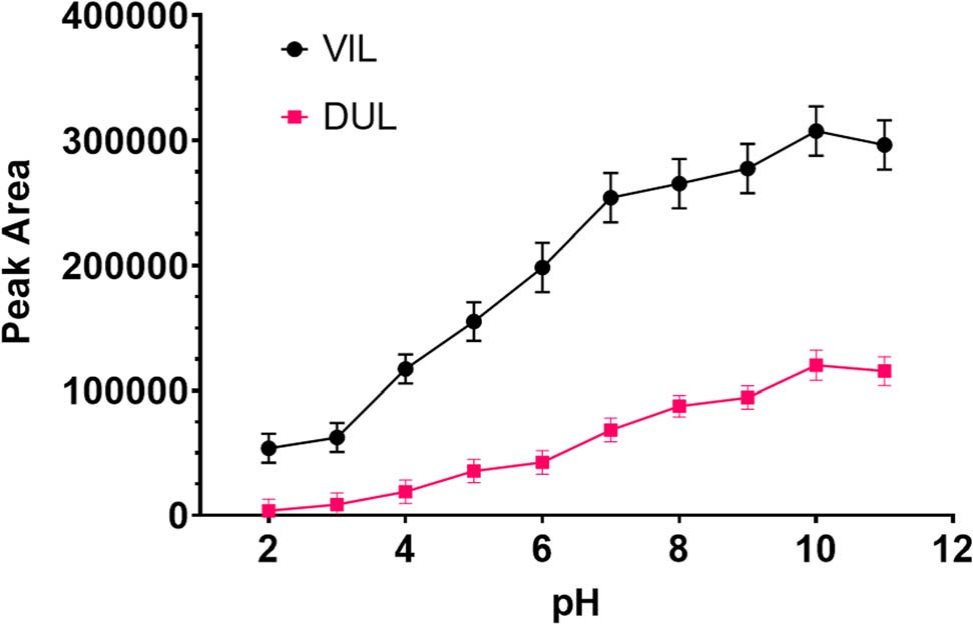
Effect of pH on the developed MSPE procedure.

The pH of the solution is one of the important factors because it affects interactions between target molecules and the surface of magnetic particles. Model solutions containing both antidepressants respectively were interacted with a series solution in the range of 2.0–11.0. Then, 50 mg of HDTMABr-coated Fe_3_O_4_ was used in all experiments. Following these processes, VIL and DUL molecules were retained on solid phase and easily separated using an external neodymium magnet. After desorption from the surface of magnetic particles using an eluent solvent, the target molecules were transferred to HPLC vials using a syringe and filtered through a 0.45 μm PTFE membrane filter. As can be seen in Fig. 6, the peak area of signals obtained with pH 10 buffer was better than that of the others. If the pKa values of VIL and DUL are 7.1 ^26^ and 9.6,^27^ respectively, then they will be in unprotonated forms in a basic medium. Therefore, pH 10 buffer was used in next step.

#### 3.2.2 Eluent type and eluent volume

**Fig. 7:**
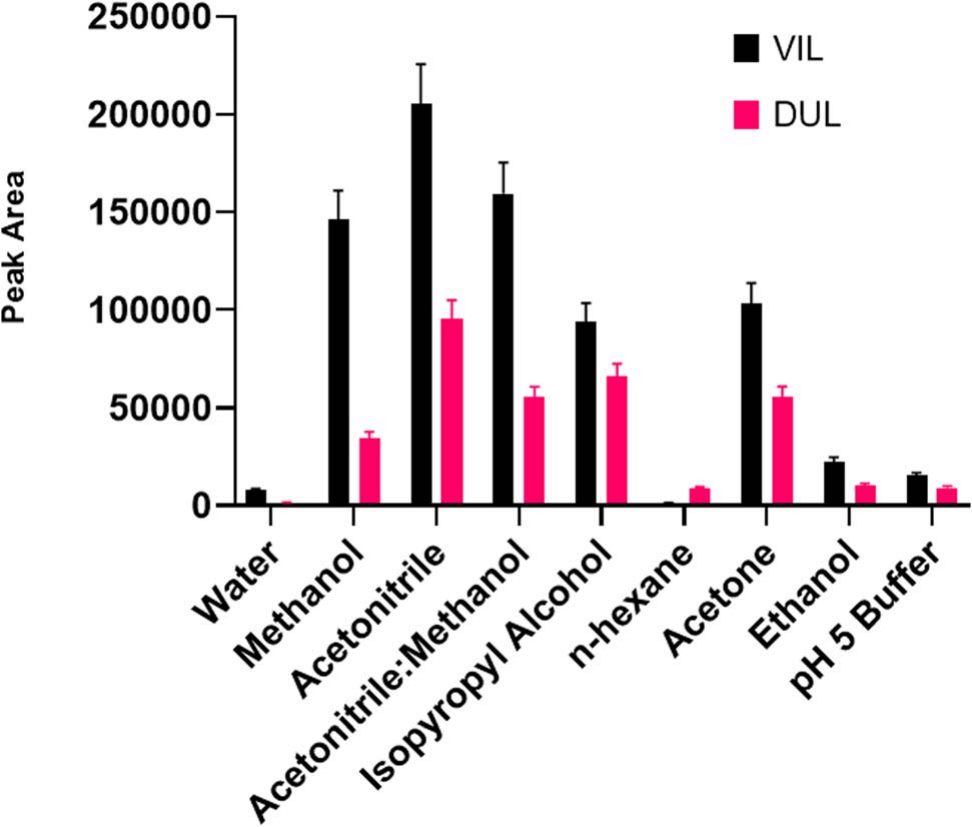
Optimization of solvent for the desorption process.

**Fig. 8:**
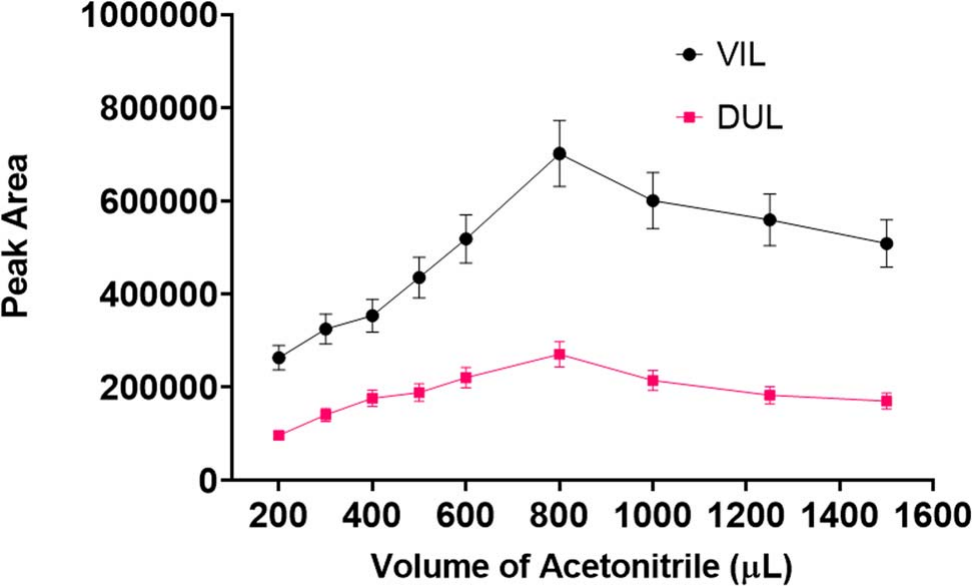
Effect of acetonitrile volume on the developed method.

The liquid phase should be removed after interactions between target molecules and solid magnetic materials were over. Generally, a simple pipet or syringe can be used for this by means of an external magnet. An ideal solvent should be suitable for the determination system and should not decompose the molecular structure of the drugs. Methanol, ethanol, acetonitrile, water, isopropanol, acetone, 50% methanol, *n*-hexane, and a mixture of acetonitrile: methanol (1 : 1, *v* : *v*) were used as desorption solvents. The experimental procedure with all steps was carried out using 1 mL of each solvent in the desorption step. As can be seen in Fig. 7, the maximum peak area was obtained with acetonitrile for both drug molecules. After the ideal solvent was determined as acetonitrile, the next optimization procedure was volume of the solvent. The final volume of desorption solvent has direct effects on the pre-concentration factor. A suitable desorption solvent should preferably be at minimal level because the pre-concentration factor is the maximal level for initial and final volumes of samples. However, it should be kept in mind that the desorption process is not performed successively with inadequate amounts of desorption solvents, and also filtration of lower volumes than 200 μL is not easy. Therefore, optimization of the volume of desorption solvent is so important for a newly developed method. The volume of acetonitrile was studied in the range of 200–1500 μL. As can be seen in Fig. 8, the highest signals were obtained with 800 μL of acetonitrile and this volume was determined as the optimal value for the desorption process.

#### 3.2.3 Optimization of time for adsorption and desorption in MSPE

**Fig. 9:**
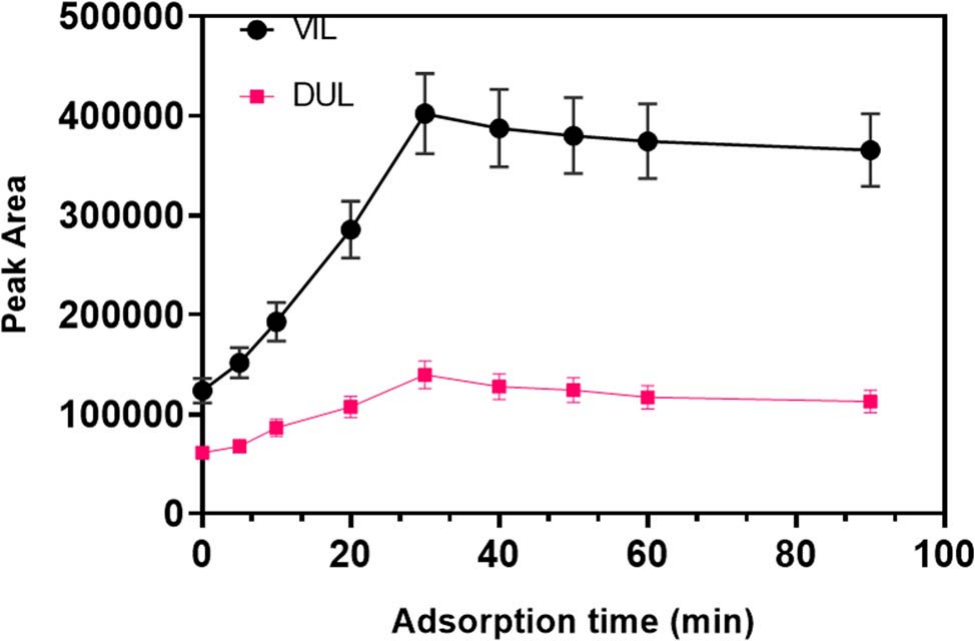
Optimization of the adsorption time.

Interactions between target molecules and solid magnetic particles are mainly based on physical and partly chemical attractions. The analysis of drug molecules was carried out in an aquatic phase and the separation of the solid phase is required after interactions were completed using an external magnet. This process can be facilitated using a kind of shaker or rotator in order to increase contact area and duration. Time of effective interactions directly related with total analysis time. Therefore, minimum time with maximum adsorption efficiency is desirable. A circular rotator was used at 50 rpm in this study in order to accelerate the experimental procedure. The optimization of time for this procedure was studied in the range of 5–90 min. As can be seen in Fig. 9, the maximum analytical signal for both molecules was obtained in 30 min of orbital time.

After the phase separation was completed in the first step, desorption of the retained molecules is generally facilitated using a simple vortex. The retained molecules on the surface of magnetic particles can be removed using a suitable solvent. The use of a vortex speeds up the transfer of drug molecules to the solvent from the surface of magnetic particles. The time of vortex procedure is also one of the optimization parameters in MSPE. Therefore, the vortex time was also optimized in the range of 5–90 s by considering the maximum peak area for both molecules. As a result of this study, it was observed that 40 s is enough for the desorption procedure.

#### 3.2.4 Reusability of the synthetized magnetic particles

The sorbents used in SPE-based methods are the most important parts of these methods. Selectivity, sensitivity, succession, durability, repeatability and cost of SPE methods are mostly dependent on the solid-phase material. Among the most important indicators for a new material are robustness and reusability. All the steps of the optimization process were performed using 50 mg of HDTMABr-coated Fe_3_O_4_ materials. The reusability of the new material was studied by using model solutions including 100 ng mL^−1^ of both drug molecules. The experiments were repeated ten times. After every use, the magnetic particles were rinsed, washed with 2 mL of acetonitrile : methanol (1 : 1; v : v) mixture, and dried in a vacuum oven at 50 °C. The evaluation of reusability was carried out by comparing the peak areas after every use. Following 10 cycles of use, the change in the peak area for VIL and DUL molecules was lower than 10% of RSD. According to experimental results, any change was observed on adsorption capability of magnetic particles for target molecules.

### 3.3. Analytical performance criteria of the method

The analytical validation of the developed MSPE-HPLC-DAD method was carried out using model solutions. Some important analytical parameters such as enhancement factor (EF), pre-concentration factor (PF), relative standard deviation (RSD), limit of detection (LOD), limit of quantification (LOQ), linear range and correlation coefficient were calculated and given in this section. The proposed MSPE-based approach was applied to model solutions containing VIL and DUL molecules at increasing concentrations in order to determine the linear working range. The linear dynamic ranges for both molecules were found to be in the range of 5.0–750.0 ng mL^−1^. The analytical signal which is considered as the peak area of target molecules in this study increases proportionally to the concentration of drug molecules throughout this range. Each point of 10 calibration values was tested in triplicate. The validation of method was carried out according to International Conference on Harmonization guidelines.^28,29^

The efficiency and success of a pre-concentration method can be expressed by comparing the analytical data before and after the proposed procedure. Moreover, a pre-concentration factor (PF) and an enhancement factor (EF) are mostly used as numerical criteria to evaluate the importance of the obtained results. MSPE experiments start with a larger volume of sample solution and a lower final volume of sample including pre-concentrated target molecules is obtained end of process. The PF value was directly calculated by the ratio of initial and final volumes of samples because the decrease in volume directly affects the increase in concentration. Therefore, the PF value was calculated as 50.0/0.8 by considering the initial and final volumes of solutions in this study. The other parameter is EF, which is calculated as the slope of calibration plots before and after MSPE. As it is well known, one of the main aims of a pre-concentration method is to increase the sensitivity. It means that it expects more analytical signal by the same determination method for a certain concentration of target molecule after the MSPE procedure. If it reaches the expected results, the slope of calibration will also increase for the same detection method. Therefore, the EF was easily calculated by using before and after slopes of calibrations.

The relative standard deviation (%RSD) presents an idea about the repeatability or precision of the method for a certain concentration. In this study, the %RSD values were calculated by using model solutions including 100 ng mL^−1^ of drug molecules for 5 replicates. As a summary of all analytical results, Table 2 is presented. It includes all calculated data by means of experimental results. It can be seen clearly in Table 2 that the advantages of the proposed method are more preferably compared with direct HPLC analysis. It is possible to make trace analysis of drug molecules at ppb level while it can be at ppm level before the MSPE.

**Table 2:** Analytical Parameters of developed method

Parameters	Before MSPE		After MSPE	
	VIL	DUL	VIL	DUL
Linear dynamic range	1,0–20,0 μg mL^−1^	1,0–20,0 μg mL^−1^	5,0–750,0 ng mL^−1^	5,0–750,0 ng mL^−1^
LOD	0,35 μg mL^−1^	0,32 μg mL^−1^	1,48 ng mL^−1^	1,43 ng mL^−1^
LOQ	0,85 μg mL^−1^	0,92 μg mL^−1^	4,71 ng mL^−1^	4,75 ng mL^−1^
RSD (%)	4,2	3,8	3,2	3,5
Calibration sensitivity	8.54	7.65	725.9	581.4
Correlation coefficient (R^2^)	0,9915	0,9965	0,9954	0,9873
Pre-concentration factor (PF)	—	—	62.5	62.5
Enhancement factor (EF)	—	—	85	76

### 3.4. Application of the developed method to real samples

The full optimization of the developed method was completed step by step in previous sections. The urine and wastewater samples are the most studied targets for antidepressant drug residues. Therefore, the proposed method was applied to these samples.

Simulated urine, urine and wastewater samples were used to test the applicability of the proposed method. The samples were both analyzed directly and also recovery tests were performed by means of spiked samples. Two different levels of target drug molecules were spiked to studied samples as 100 and 300 ng mL^−1^. Any concentration of VIL and DUL molecules were determined in the samples. In the spiked samples, %recovery and %RSD values were calculated by using the experimental results of three replicate samples. As shown in Table 3, %recoveries in the range 95.7 and 104.8 and %RSDs in the range 3.4 and 5.2 were obtained by the application of the proposed method. These results provided that the accuracy and precision of this method are acceptable.

**Table 3:** Application of the proposed method to samples

Samples	Added ng mL^−1^	Found^a^ ng mL^−1^		RSD %		Recovery %	
		VIL	DUL	VIL	DUL	VIL	DUL
Wastewater	0.0	<LOD	<LOD	—	—	—	—
	100,0	95,7 ± 4,1	103,2 ± 3,5	4,3	3,4	95,7	103,2
	300,0	295,1 ± 12,9	302,5 ± 14,5	4,4	4,8	98,3	100,8
Situmulated urine	0.0	<LOD	<LOD	—	—	—	—
	100,0	99,7 ± 3,9	104,8 ± 3,9	3,9	3,7	99,7	104,8
	300,0	290,9 ± 14,1	297,0 ± 12,5	4,8	4,2	96,7	99,0
Urine	0,0	<LOD	<LOD	—	—	—	—
	100,0	99,8 ± 3,7	104,8 ± 5,5	3,7	5,2	99,8	104,8
	300,0	296,7 ± 13,2	308,4 ± 16,0	4,4	5,2	99,2	102,8

a _The average value of five replicates ± standard deviation (*N* = 5)_.

## 4. Conclusions

Solid-phase-based approaches as pre-treatment methods are desirable due to easy applicable sides for every type of samples. The combination of magnetic solid-phase extraction and HPLC-DAD system presents a very useful tool for the determination of drug molecules in biological matrices. The use of magnetic materials as sorbents in SPE has important advantages such as easy and fast separation of solid materials, and surface modification depending on target molecules.

The sensitive determination of VIL and DUL drug molecules was carried out by means of newly synthetized magnetic nanoparticles. The determination step was performed by using the HPLC-DAD system. The use of hexadecylammonium bromide for the surface modification of magnetic particles provided both hydrophobic and electrostatic interactions together for target drug molecules. Thanks to the developed method, it is possible to determine VIL and DUL molecules at ng mL^−1^ (ppb) level, while their direct determination with the HPLC-DAD system provides this analysis at μg mL^−1^ (ppm) level.

The developed method presents simple operation, high sensitivity, wide linearity, and excellent recovery for the target antidepressant molecules. The most important advantage of the proposed method is the ability to determine these molecules at very low levels by conventional HPLC, which is easily accessible for most laboratories. However, the analysis of these molecules needs complex, hybrid and expensive systems like HPLC-MS.

## CRediT authorship contribution statement

Halil İbrahim Ulusoy: Investigation, writing – original draft, data curation, supervision, project administration, funding acquisition, validation. Ümmügülsüm Polat: Formal analysis, formal analysis, data curation, validation. Songül Ulusoy: Conceptualization, writing – original draft, writing – review & editing.

## Data availability

Data will be made available on request.

## Conflicts of interest

The authors declare that they have no known competing financial interests or personal relationships that could have appeared to influence the work reported in this paper.

## Supplementary Material
